# Elective Tracheotomy in Patients Receiving Mandibular Reconstructions: Reduced Postoperative Ventilation Time and Lower Incidence of Hospital-Acquired Pneumonia

**DOI:** 10.3390/jcm12030883

**Published:** 2023-01-22

**Authors:** Johannes G. Schuderer, Leonie Reider, Michael Wunschel, Gerrit Spanier, Steffen Spoerl, Maximilian Josef Gottsauner, Michael Maurer, Johannes K. Meier, Peter Kummer, Torsten E. Reichert, Tobias Ettl

**Affiliations:** 1 Department of Oral and Maxillofacial Surgery, University Hospital Regensburg, 93053 Regensburg, Germany; 2 Section Phoniatrics and Pediatric Audiology, Department of Otolaryngology, University Hospital Regensburg, 93053 Regensburg, Germany

**Keywords:** reconstructive surgery, tracheotomy, microvascular reconstruction, mandibular reconstruction, airway management, hospital acquired pneumonia

## Abstract

Elective tracheotomy (ET) secures the airway and prevents adverse airway-related events as unplanned secondary tracheotomy (UT), prolonged ventilation (PPV) or nosocomial infection. The primary objective of this study was to identify factors predisposing for airway complications after reconstructive lower ja surgery. We reviewed records of patients undergoing mandibulectomy and microvascular bone reconstruction (N = 123). Epidemiological factors, modus of tracheotomy regarding ET and UT, postoperative ventilation time and occurrence of hospital-acquired pneumonia HAP were recorded. Predictors for PPV and HAP, ET and UT were identified. A total of 82 (66.7%) patients underwent tracheotomy of which 12 (14.6%) were performed as UT. A total of 52 (42.3%) patients presented PPV, while 19 (15.4%) developed HAP. Increased operation time (OR 1.004, *p* = 0.005) and a difficult airway (OR 2.869, *p* = 0.02) were predictors, while ET reduced incidence of PPV (OR 0.054, *p* = 0.006). A difficult airway (OR 4.711, *p* = 0.03) and postoperative delirium (OR 6.761, *p* = 0.01) increased UT performance. HAP increased with anesthesia induction time (OR 1.268, *p* = 0.001) and length in ICU (OR 1.039, *p* = 0.009) while decreasing in ET group (HR 0.32, *p* = 0.02). OR for ET increased with mounting CCI (OR 1.462, *p* = 0.002) and preoperative radiotherapy (OR 2.8, *p* = 0.018). ET should be strongly considered in patients with increased CCI, preoperative radiotherapy and prolonged operation time. ET shortened postoperative ventilation time and reduced HAP.

## 1. Introduction

Perioperative airway management in patients undergoing complex head and neck reconstruction is fundamental. Airway impairment due to postoperative flap swelling, oedema or hematoma is a feared side effect following oromandibular resection and immediate reconstruction [1,2]. Perioperative elective tracheotomy secures the airway and prevents adverse airway-related events such as unplanned secondary tracheotomy, prolonged ventilation or nosocomial infection [3,4,5]. Attempts are made to reduce the cannulation time, the overall length of hospitalization and tracheotomy-associated complications such as bleeding, emphysema or functional impairment [6]. Despite efforts to establish objective criteria for elective tracheotomy in mandibular reconstruction, airway strategies are primarily based on individual risk evaluation and the surgeons’ expertise [6,7,8,9]. Therefore, to establish more objective benchmarks, the primary objective of this study was to identify factors predisposing for airway complications comprising prolonged postoperative ventilation >48 h and hospital acquired pneumonia (HAP). The occurrence of elective and unplanned tracheotomies (UT) were secondary endpoints.

## 2. Materials and Methods

We reviewed records of patients who underwent mandibulectomy and microvascular bone reconstruction at the Clinic of the University of Regensburg between 2006 and 2018. For all cases, we recorded patient-specific characteristics, including age, gender, diagnosis, previous radiation, neck dissection, classification of mandibular resection defects (Brown classification [10]), number of graft segments, flap and donor site complications, duration of surgery and anaesthesia induction time, as well as general medical conditions. The Charlson Comorbidity Index [11] was used to determine overall disease severity. In addition, we analyzed the total duration of hospitalizations, the amount of time spent in the intensive care unit, normal ward and the duration of postoperative ventilation. Additionally, we recorded the occurrence of difficult airways, as defined by the American Society of Anaesthesiologists “as the clinical situation in which a conventionally trained anaesthesiologist experiences difficulty with facemask ventilation of the upper airway, difficulty with tracheal intubation, or both” [12]. The occurrence of emphysema, bleeding or infection were defined as complications at tracheostoma sites. We classified the type of tracheotomy as follows: if the tracheotomy was performed perioperatively alongside reconstructive surgery, it was considered as elective. By contrast, if it was performed postoperatively during inpatient stay, it was considered unplanned (UT). Most UTs were performed under controlled conditions in the OR due to difficulties or risks of extubation after long-term intubation in ICU. In very rare cases, a bedside emergency tracheostomy was necessary in ICU due to insufficient spontaneous patient breathing after extubation and failure of re-intubation. There was no differentiation possible in retrospective settings.

Flap success was defined as complete healing without loss of flap integrity in the recipient area. Partial loss was defined as detachment of skin layers or skin islands in postoperative course due to vessel issues or surgical site infections. Flap revision was considered a surgical intervention with exploration of anastomosis due to flap crisis. Both partial flap loss and minor wound healing disturbances or dehiscences were defined as flap complications.

To wean patients from tracheotomy, the cuff was deflated for at least 24 h while blood oxygenation and expectoration were closely monitored. Afterward, phoniatric dysphagia diagnostic was performed using fiberoptic endoscopic evaluation of swallowing (FEES). If aspiration was inapparent, a speech cannula was inserted, and the stoma was surgically closed several days later.

We recorded the postoperative ventilation time and determined whether patients suffered from hospital-acquired pneumonia (HAP) upon their discharge. Patients were diagnosed with HAP either when they presented with a new infiltrate in their chest X-ray or purulent sputum in combination with elevated body temperature of over 38 °C [13]. Postoperative ventilation >48 h or HAP were determined to be primary endpoints, while elective tracheotomy and occurrence of UT were considered secondary endpoints.

The study was conducted under local ethical committee approval for retrospective studies conducted in accordance with ethical standards of the Declaration of Helsinki.

### Statistical Analysis

Univariate statistics were performed using Fisher’s exact test, the Chi-squared test and Student’s T-test, which were chosen based on the size of the respective reference groups. Multivariate logistical regression analysis was performed to identify predictors for UT, HAP and overall tracheotomy after adjusting for patients’ characteristics, comorbidities and surgical features. In our initial model, we included age, sex, diagnosis, localization, previous surgery, difficult airway, neck dissection, Brown classification, number of bone segments, occurrence of delirium, duration of surgery, previous radiotherapy, length of hospitalization on normal ward and intensive care unit and Charlson Comorbidity Index. A conclusive model was then constructed from variables that were found to be significant predictors for UT, HAP and overall tracheotomy. Using the maximum likelihood method, we computed regression coefficients (B) and odds ratios (OR). Subsequently, 95% confidence intervals (CI) were calculated, and *p* ≤ 0.05 was considered to be statistically significant. In our univariate analysis, only significant *p*-values were displayed. A Cox proportional hazards regression model was used to calculate the risk of HAP during hospital stay with regard to elective tracheotomy (by using log rank test for significance assessment). SPSS version 26.0 (IBM Corp.) was used.

## 3. Results

We included 123 patients in our study that underwent microvascular bone reconstruction following mandibulectomy. In all cases, segmental mandibular resection was performed prior to reconstructive surgery. A total of 82 patients (67%) underwent tracheotomy, of which 12 were unplanned (15%). A total of 52 patients (42%) had prolonged ventilation time >48 h. Nineteen patients (15%) developed HAP. Patient characteristics are presented in Table 1, Table 2 and Table 3.

The average age of our patient population was 57 ± 12.7 years; 42 patients were female (34.1%), and 81 patients were male (65.9%). Overall, patients had an average Charlson Comorbidity Index of 3.1 ± 2.0 points. The most common cause for mandibulectomy was oral tumors (73.2%), followed by radioosteonecrosis (RONJ, 19.5%) and medication-related osteonecrosis (MRONJ, 2.4%). A total of 73 patients (59.3%) had previously undergone head and neck surgery, while 52 (42.5%) had already undergone radiation therapy by the time of reconstruction. Difficult airway was diagnosed in 44 cases (35.8%), and in 89 patients (72.4%), a neck dissection was performed. The most common postoperative defect type was Brown class III (44.7%), followed by II (25%). Microvascular bone reconstruction was performed using Fibula in 110 cases (89.4%), followed by iliac bone graft in eleven cases (DCIA 8.9%) and Scapula in two cases (1.6%). On average, 2 ± 0.8 bone segments were reconstructed. On average, reconstructive surgery lasted 540 ± 156 min after 70 ± 23 min of anaesthesia induction. Patients were hospitalized for 21.4 ± 0.8 days on average, of which they spent 4.8 ± 4.7 days on the intensive care unit and 17.1 ± 9.1 days on the normal ward.

Univariate analysis showed that patients who were ventilated postoperatively for more than 48 h (prolonged ventilation group) underwent unplanned tracheotomy significantly more often than patients with postoperative ventilation for less than 48 h (regular ventilation group; 21.2% vs. 1.4%, respectively; *p* < 0.001). As expected, patients in the prolonged ventilation group also required more time until the tracheostoma was closed, when compared with patients in the regular ventilation group (23.6 days vs. 15.1 days, respectively; *p* = 0.03), and oral intake was postponed (18.1 days vs. 12.1 days; *p* = 0.04). Furthermore, we could associate prolonged ventilation with flap revision (30.8% vs. 5.6%; *p* < 0.001), flap related complications (67.3% vs. 49.3%; *p* = 0.05) and decreased flap success (90.1% vs. 76.9%; *p* = 0.05).

In addition, prolonged ventilation, in comparison to regular ventilation, was associated with an increased incidence of postoperative delirium (27% vs. 7%, respectively; *p* = 0.003), incidence of HAP (23.1% vs. 10%; *p* = 0.04), total duration of inpatient stay (25.1 days vs. 18.7 days; *p* < 0.001) and prolonged stay on the intensive care unit (8 days vs. 2.3 days; *p* < 0.001) (Table 1).

Our univariate analysis showed that the development of HAP was more frequent in patients with difficult airways when compared with the remaining patients (57.9% vs. 31.7%, respectively; *p* = 0.03) and in patients whose surgeries involved reconstruction of an increased number of bone segments (2.4 vs. 1.9, respectively; *p* = 0.03). Patients with HAP developed delirium more frequently than patients without HAP (36.8% vs. 11.5%, respectively; *p* = 0.005), required longer anaesthesia induction times (80.2 min vs. 69.5 min; *p* = 0.03), were ventilated postoperatively for longer durations (4.3 days vs. 2.7 days; *p* = 0.001) and were hospitalized longer on our intensive care unit (7.6 days vs. 4.8 days; *p* < 0.001) (Table 1).

Patients who underwent tracheotomy were older on average when compared to patients that did not receive tracheotomy (59.1 years vs. 52.9 years; *p* = 0.01) and had predominantly experienced primary (tumor) surgery more often (80.5% vs, 48.8%, respectively; *p* = 0.001). Patients that underwent neck dissection required tracheostomy more often than the remaining patients (81.7% vs. 53.7%, respectively; *p* = 0.001). Unsurprisingly, patients receiving Brown class III resections were tracheotomized more often when compared to the remaining patients (55% vs. 26.8%, respectively; *p*= 0.04), whereas patients with Brown class I resections required fewer tracheotomies when compared to the remaining patients (8.8% vs. 34.1%, respectively; *p* < 0.001). CCI averaged higher in the tracheotomy group compared to patients that did not require tracheotomy (3.6 vs. 2.2, respectively; *p* < 0.001).

When comparing both tracheotomy styles with univariate analysis, we found that closure of tracheotomy sites was delayed in UT compared to elective procedures (28.6 days vs. 17.3 days, respectively; *p* = 0.03), that there was an increased incidence of difficult airway in UT (58.3% vs. 25.7%; *p* = 0.02) and an increase in postoperative delirium in UT (50% vs. 18.6%; *p* = 0.02). Patients undergoing UT had longer ventilation times (5.7 days vs. 2.7 days, respectively; *p* < 0.001). Hospitalization on the intensive care unit averaged 9.7 days for patients with UT (*p* < 0.001). Because patients with UT spent more time overall in the intensive care unit, their hospitalization in the normal ward was shorter when compared with patients undergoing elective tracheotomy (14.8 days vs. 21 days, respectively; *p* = 0.03) (Table 2).

Patients undergoing tracheotomy were older on average when compared to patients that did not receive tracheotomy (59.1 vs. 52.9 years, respectively; *p* = 0.01), were ranked with a higher Charleson point score (3.6 vs. 2.2; *p* < 0.001) and had to undergo neck dissection more often (81% vs. 54%; *p* = 0.001). Following surgeries, the resulting mandibular resection defects were ranked significantly more frequently as Brown class III in the tracheotomy group (44 vs. 11 respectively; *p* = 0.04) and significantly less frequently as Brown class I in the no-tracheotomy group (14 vs. 7, respectively; *p* < 0.001). In addition, tracheotomy was associated with reconstructions requiring more bone segments (mean 2.1) compared to the non-tracheotomy group (mean 1.7; *p* = 0.01). Flap success rate was higher (95.1%) in the non-tracheotomy group compared to the patients who received planned or unplanned tracheotomy (79.3%, *p* = 0.02), while complications were significantly lower when no tracheotomy was performed (*p* = 0.001). Likewise, surgeries in the tracheotomy group were significantly longer compared to the non-tracheostomy group (587 vs. 446 min, respectively; *p* < 0.001), and also their hospitalization on the intensive care unit (*p* = 0.05) and the normal ward (*p* < 0.001) was significantly extended (Table 3).

Multivariate regression showed that increased operation time (OR 1.004, *p* = 0.005, CI = 1.001–1.007) and difficult airways (OR 2.869, *p* = 0.02, CI = 1.161–7.088) were predictors for prolonged postoperative ventilation, while elective tracheotomy reduced the need for prolonged ventilation (OR 0.054, *p* = 0.006, CI = 0.007–0.440). The presence of a difficult airway (OR 4.711, *p* = 0.03, CI = 1.145–19.389) and postoperative delirium (OR 6.761, *p* = 0.01, CI = 1.594–29.229) increased the chance for UT. An increased anaesthesia induction time (OR 1.268, *p* = 0.001, CI = 1.101–1.461) and prolonged hospitalization on the intensive care unit (OR 1.039, *p* = 0.009, CI = 1.009–1.069) were predictors for HAP occurrence (Table 4).

**Figure 1:**
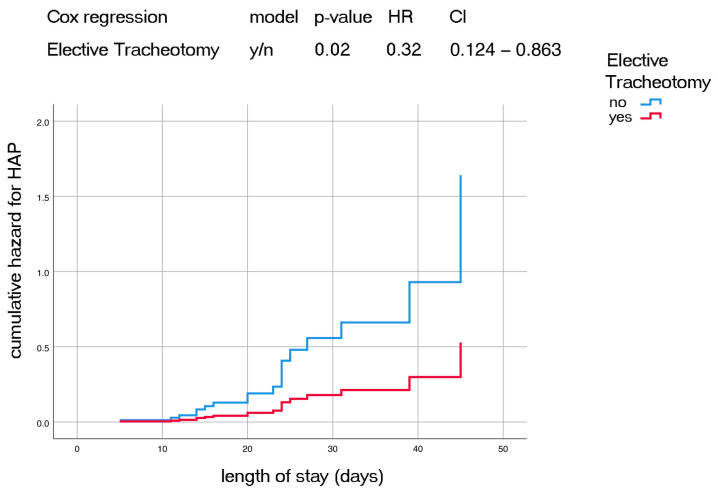
Cox Regression HR function for HAP in elective tracheotomy patients over total length of stay. HAP= hospital acquired pneumonia; HR = hazard ratio, CI = confidence interval.

We investigated the occurrence of HAP in patients with elective tracheotomy using Cox regression analysis for hazard ratio (Figure 1, Table 4) and found that the risk for developing HAP decreased for patients with elective tracheotomy over total length of hospitalization (HR 0.32, *p* = 0.02, CI = 0.124–0.863).

The odds ratio for having an elective tracheotomy increased with mounting CCI (OR 1.462, *p* = 0.002, CI= 1.152–1.854) and preoperative radiotherapy (OR 2.8, *p* = 0.018, CI = 1.184–6.257). When investigating the outcomes of different mandibular defect types, we found that Brown class III (OR 2.646, *p* = 0.038, CI = 1.055–6.637) increased the probability for elective tracheotomy. Multivariate regression showed that both difficult airway (OR 4.711, *p* = 0.03, CI = 1.145–19.389) and occurrence of postoperative delirium (OR = 6.761, *p* = 0.01, CI = 1.594–29.229) were predictors for unplanned tracheotomy.

## 4. Discussion

To avoid airway complications, elective tracheotomy is a standard surgical procedure for patients undergoing reconstruction of oral and mandibular defects. Thanks to the constant evolution of microvascular reconstruction techniques, the durations of surgery and hospitalization continue to decrease, while flap success rates increase. However, appropriate airway management remains challenging [5]. It should be a priority to keep the postoperative in-patient time short and focus on the recovery of physiological functions, such as oral intake and swallowing [14]. However, recommendations for mandibular reconstruction vary and definite guidelines for elective tracheotomy and their implementation are pending [6,15,16,17]. Among others, Gupta et al. (2016) defined the CASST score, which is a set of criteria based on surgical and epidemiological factors that facilitates the decision for elective tracheotomy in patients that underwent head and neck surgery [16].

The major criteria were: previously radiated in same region of surgery, resection of two more sub-sites of oral cavity or oropharynx, bilateral neck dissection, extended hemi or central arch mandibulectomy, bulky flap for reconstruction: latissimus dorsi; double skin island pectoralis major myocutaneous flap, flap with a compressing element: intact mandibular rim; use of a concomitant reconstruction plate.

However, due to a low specificity of 90% and sensitivity of 70%, patients that should receive elective tracheotomy are frequently overlooked when relying solely on their CASST score. Thus, the medical expertise and subjective experience of the surgeons remain important factors in this decision [16,17].

Some authors suggest delayed extubation instead of elective tracheotomy as a less invasive method for securing the airways [15]. However, delayed extubation is a well-investigated risk factor for the development of hospital-acquired pneumonia and delirium [18,19,20]. By contrast, it has been shown that early postoperative extubation decreases the duration of the overall hospitalization and on the intensive care unit.

Our data suggested that both difficult airway and an increasing duration of surgery were predictive markers for prolonged postoperative ventilation and unplanned secondary tracheotomy. In addition, elective tracheotomy appeared to reduce the likelihood of prolonged postoperative ventilation. To prevent postoperative airway complications, Xu et al. developed a prediction algorithm that determines the necessity for elective tracheotomy [21]. Among others, preoperative radiation was found to be a risk factor for airway distress in this study. In our study, 42% of patients received preoperative radiation, of which 31 underwent mandibular resection due to osteoradionecrosis. We found that previous radiation exposure in the head and neck area was an independent predictor for elective tracheotomy in our study group. Radiation above 60 Gray resulted in clinically manifested fibrosis, which, in turn, may significantly impair bone nutrition and result in infections, pathological fractures and various other postoperative complications [22,23]. Restricted neck movement, trismus and muscle-relaxant-resistant altered pharynx physiology can complicate laryngoscopy (both direct and via video observation), thus resulting in a difficult airway [24,25]. Deng et al. showed that radiotherapy was associated with persistent lymphedema of the upper aerodigestive tract in up to 65% of patients with a history of additive therapy following head and neck cancer [26]. By considering our results in the context of the work of Cameron et al., Kruse-Lösler et al. and Xu et al., the combination of altered anatomy, postoperative flap swelling and additional lymphedema could be explained [17,21,27]. The latter aggravates the occurrence of adverse airway events, such as delayed extubation, during perioperative intubation. This highlights the necessity for elective tracheotomy in this patient group.

With an incidence of up to 50%, HAP is ranked as the most common complication following major head and neck surgeries [28]. The most prominent risk factors for HAP after head neck surgery are a postoperative ventilation duration greater than 48 h and the time spent on the intensive care unit [22]. Postoperatively, the loss of protective reflexes can promote aspiration of saliva and thus lead to the development of HAP unattached to tracheotomy status [29]. This problem is aggravated by prolonged surgery and mechanical ventilation [30,31]. In our cohort, the development of HAP was significantly associated with prolonged anaesthesia induction time and a longer stay on the intensive care unit. The latter finding is confirmed by the work of Xu et al., who showed that the risk for HAP increased with the duration of postoperative hospitalization [31].

Several studies reported that elective tracheotomy is a predictor for HAP development [6,31,32]. Li et al. for example, showed that canulation for more than 20 days was associated with HAP [32]. In our cohort, the mean duration of canulation was only 12.6 days, suggesting that canulation was not a main reason for HAP in our study.

Our data showed that the hazard ratio for HAP during the hospitalization period was lower in the elective tracheotomy group. This is supported by the data of Loeffelbein et al., who showed that patients undergoing head and neck surgery and who had been tracheotomized intraoperatively, had a reduced risk (by a factor of 2.4) for pulmonary complications [30]. Meier, Leiser and Georges et al. found that early elective tracheotomy shortens the duration of sedation and the time spent on the intensive care unit, which are both inherent risk factors for HAP [3,4,33].

Additionally, it has been shown that intensified oral hygiene can have a protective effect against the development of pneumonia. However, oral colonization with certain respiratory bacteria strains that are resident in oral biofilm may also serve as an infective reservoir [34,35]. While tracheostomas are easy to clean, mechanical ventilation through a tracheal tube provides an entryway for bacterial invasion into the upper airways. By contrast, tracheotomy allows for increased oral hygiene and can thus reduce the incidence of postoperative HAP on intensive care units [28].

Moubayed and Lin et al. conducted the first retrospective study of patients who did not undergo elective tracheotomy after mandibular reconstruction. Although there was no statistical control group in their study, they found that central mandibular defects would necessitate elective tracheotomy [7,8]. This is in accordance with our data, which shows that resection of the mandibular midline (resulting in a Brown III classification) increased the odds for elective tracheotomy by a factor of three. Tracheotomy in these cases is necessary despite refixation of the suprahyoid muscles to the reconstruction plate or the graft, which was performed in every patient using 2.0 permanent or PDS sutures. In our experience, it takes several days to weeks until the patient becomes accustomed to this new positioning of his tongue. Xu et al. developed a decision algorithm that recommended whether patients with free flap reconstruction and mandibulectomy with midline crossing should receive an elective tracheotomy [21].

Various studies were able to show that neck dissection was a positive predictor of postoperative airway obstruction [17,21]. Bilateral neck dissection is reflected in the CASST Score by Gupta et al. as predictor for the need of a tracheostomy [16]. In contrast, there is no clear statement in the literature regarding the need for a tracheotomy in the case of unilateral neck dissection. In our study group, significantly more patients with neck dissection were tracheotomized compared to the control group (*p* = 0.001). However, the independent variable lost significance under multivariate regression. With regard to neck dissection, one limitation in this study is that the effect of neck dissection could not be further specified as exact data for type of neck dissection (unilateral vs. bilateral, elective vs. therapeutic, and dissection levels) were not available.

The Charlson Comorbidity Index (CCI) appeared to be a normative predictor under controlled regression and showed that the odds for elective tracheotomy were increased by 1.3 in our study group. The CCI and its adjusted versions are frequently used for survival prognosis and disease severity assessment in patients with head and neck cancer [36,37,38].

Presumably, multimorbidity directly affects the postoperative outcomes of different airway management strategies. Therefore, an experienced surgeon should carefully assess pre-existing medical conditions to determine whether to perform elective tracheotomy and which ventilation modality to apply.

Furthermore, we found that flap success was reduced from around 91% to 77% in the group with prolonged ventilation and overall in patients with elective tracheotomy. It can be assumed that overall flap success is moderated by many factors. First of all, one can assume that patients with a flap crisis spend longer in the ICU and are exposed to an increased risk of swelling due to a second intervention. This might increase the chance for unplanned tracheotomy as the CASST score indicates [16]. Additionally, patients who had undergone elective tracheostomy scored higher in their Charleson score as our multivariate models suggests (Table 3). As Katna et al. display, patients with cardiovascular predisposition and poor vessel status who scored high in CCI presented a higher perioperative event rate, moderating microvascular flap success [39].

A common complication after inpatient surgery is delirium. Some authors suggest that the incidence of delirium is about 15%, which is consistent with our data [3,40].

We found that patients with HAP or unplanned tracheotomy showed postoperative delirium significantly more often. In our study, the chance of being secondarily tracheostomized was increased by a factor of 6.7 in patients who showed signs of delirium. A recent multicenter study by Gazda et al. associated early tracheotomy with lower odds of postoperative delirium [41]. While age seems to be a prominent risk factor for delirium, elective tracheotomy shortens the hospitalization on intensive care unit and shortens ventilation times, which in turn reduces the risk for delirium [3,42].

This study has some limitations due to its retrospective design. In principle, it cannot be ruled out that there was a selection bias for which patients received an elective tracheotomy, because surgeons made that decision subjectively on the basis of their previous experience. In addition, internal agreements between the Department of Surgery and Department of Anaesthesia contributed to certain postoperative processes. Nevertheless, this study is currently the largest study that approaches tracheotomy exclusively in mandibular defects with bone reconstruction and our results should help to address postoperative airway management.

## 5. Conclusions

Elective tracheotomy should be strongly considered in patients with increased Charleson Comorbidity Index preoperative radiotherapy, prolonged surgery duration and midline-crossing mandibular defects. Elective tracheotomy can also shorten the duration of postoperative ventilation and reduce the incidence of hospital-acquired pneumonia. Patients with pre-surgical difficult airway and prolonged surgeries have a predisposition for prolonged postoperative ventilation, deliriant state and the need for a secondary tracheotomy.

## Figures and Tables

**Table 1:** Patient and treatment characteristics associated with postoperative ventilation time >48 h and hospital acquired pneumonia.

		Ventilation ≤ 48 h N = 71	Ventilation > 48 h N = 52	*p*-Value	No HAP N = 104	HAP N = 19	*p*-Value
Sex	Men	45 (63.4%)	36 (69.2%)		70 (67.3%)	11 (57.9%)	
	Women	26 (36.6%)	16 (30.8%)		34 (32.7%)	8 (42.1%)	
Age	years	57.3 ± 13.9	56.7 ± 10.9		56.7 ± 13.1	58.7 ± 9.9	
Previous surgery head neck	Yes	43 (60.6%)	30 (57.7%)		60 (57.7%)	13 (68.4%)	
Diagnosis							
	Tumor	44 (62%)	35 (67.3%)		71 (68.3%)	8 (42.1%)	
	RONJ	16 (22.5%)	15 (28.8%)		23 (22.1%)	8 (42.1%)	
	MRONJ	2 (2.8%)	1 (1.9%)		2 (1.9%)	1 (5.3%)	
	Osteomyelitis	8 (11.3%)	1 (1.9%)		7 (6.7%)	2 (10.5%)	
	Trauma	1 (1.4%)			1 (1%)		
Overall Tracheotomy	Yes	45 (63.4%)	37 (71.2%)		68 (65.4%)	14 (73.7%)	
Elective tracheotomy	Yes	44 (62%)	26 (50%)		60 (57.7%)	10 (52.6%)	
Unplanned tracheotomy	Yes	1 (1.4%)	11 (21.2%)	<0.001	8 (7.7%)	4 (21.1%)	
No tracheotomy	Yes	26 (36.6%)	15 (28.8%)		36 (34.6%)	5 (26.3%)	
Closure of tracheotomy	days	15.1 ± 10.2	23.6 ± 19.1	0.03	18.8 ± 15.9	21.6 ± 14.1	
Complication Tracheostoma site	Yes	10 (14.1%)	11 (21.2%)		17 (16.3%)	4 (21.1%)	
Difficult airway	Yes	23 (32.4%)	21 (40.4%)		33 (31.7%)	11 (57.9%)	0.03
Oral intake after surgery	days	12.7 ± 6.5	18.1 ± 7.3	0.04	15.1 ± 6.8	15.8 ± 8.7	
Neck dissection	Yes	47 (66.2%)	42 (80.8%)		76 (73.1%)	13 (68.4%)	
Brown Classification							
	I	13 (18.3%)	8 (15.4%)		19 (18.3%)	2 (10.5%)	
	Ic	1 (1.4%)	3 (5.8%)		3(2.9%)	1 (5.3%)	
	II	21 (29.6%)	9 (17.3%)		28 (26.9%)	2 (10.5%)	
	IIc	1 (1.4%)	3 (5.8%)		3 (2.9%)	1 (5.3%)	
	III	29 (40.8%)	26 (50%)		45 (43.3%)	10 (52.6%)	
	IV	5 (7%)			4(3.8%)	1 (5.3%)	
	IVc	1 (1.4%)				1 (5.3%)	
Flap type							
	Fibula	64 (90.1%)	46 (88.5%)		92 (88.5%)	18 (94.7%)	
	DCIA	6 (8.5%)	5 (9.6%)		10 (9.6%)	1 (5.3%)	
	Scapula	1 (1.4%)	1 (1.9%)		2 (1.9%)		
Bone Segments	n	1.9 ± 0.8	2.1 ± 0.8		1.9 ± 0.8	2.4 ± 0.7	0.03
Flap Revision	Yes	4 (5.6%)	16 (30.8%)	<0.001	17 (16.3%)	3 (15.8%)	
Flap success	Yes	64 (90.1%)	40 (76.9%)	0.05	87 (83.7%)	17 (89.5%)	
Partial Flap Loss	Yes	23 (32.4%)	19 (36.5%)		35 (33.7%)	7 (36.8%)	
Donor Site Complication	Yes	17 (23.6%)	16 (30.8%)		30 (28.8%)	3 (15.8%)	
Flap Site Complication	Yes	35 (49.3%)	35 (67.3%)	0.05	60 (57.7%)	10 (52.6%)	
Delirium	Yes	5 (7%)	14 (26.9%)	0.003	12 (11.5%)	7 (36.8%)	0.005
Anaesthesia induction time	Ø min	73.2± 22.8	64.5 ± 23.1	0.04	67.6 ± 23	80.2 ± 22.2	0.03
Length of surgery	Ø min	504 ± 120.3	589.9 ± 184.6		541.5 ± 149.9	533.6 ± 190.7	
Preoperative Radiotherapy	Yes	30 (42.3%)	22 (42.3%)		42 (40.4%)	10 (52.6%)	
Cumulative dose	Gray	62.2 ±9.5	64.9 ± 7.5		62.9 ± 8.5	64.7 ± 11.1	
Normal ward	days	16.4 ± 8.5	18.1 ± 10		17.4 ± 8.5	15.4 ± 12.3	
Intensive Care Unit	days	2.3 ± 1.1	8 ± 5.6	<0.001	3.9 ± 3.6	7.6 ± 6.3	<0.001
Length of stay	days	18.7 ± 8.6	25.1 ± 9.6	<0.001	21.1 ±9.1	23.1 ±2.6	
Postoperative ventilation time	days	1.8 ± 0.4	4.6 ± 1.8		2.7 ± 1.6	4.3 ± 2.6	0.001
Ventilation > 48 h	y/n				40 (38.5%)	12 (63.2%)	
BMI	kg/m^2^	24.2 ± 4.7	23.3 ± 4.5		23.6 ± 4.3	24.7 ± 6.4	
Smoking	Yes	49 (69%)	38 (73.1%)		73 (70.2%)	14 (73.3%)	
Alcohol	Yes	37 (52.1%)	32 (61.5%)		60 (57.7%)	9 (47.4%)	
Coronary heart disease	Yes	7 (9.9%)	6 (11.5%)		11 (10.6%)	2 (10.5%)	
Hypertension	Yes	32 (45.1%)	22 (42.3%)		44 (42.3%)	10 (52.6%)	
Charlson Comorbidity Index	Points	2.9 ± 1.9	3.33 ± 2.1		3.1 ± 2	3 ± 1.9	
COPD	Yes	8 (11.3%)	8 (15.4%)		13 (12.5%)	3 (15.8%)	
HAP	Yes	7 (9.9%)	12 (23.1%)	0.04			

**Table 2:** Patient and treatment characteristics associated with elective and unplanned tracheotomy.

		Elective Tracheotomy Yes N = 70	Unplanned Tracheotomy Yes N = 12	*p*-Value
Sex	Men	46 (65.7%)	8 (66.7%)	
	Women	24 (34.3%)	4 (33.3%)	
Age	Ø years	59.5 ± 10.8	56.6 ± 13.4	
Previous surgery head neck	Yes	32 (45.7%)	8 (66.7%)	
Diagnosis				
	Tumor	53 (75.7%)	7 (58.3%)	
	RONJ	12 (17.1%)	4 (33.3%)	
	MRONJ	1 (1.4%)	1 (8.3%)	
	Osteomyelitis	4 (5.7%)		
	Trauma	-		
Closure of tracheotomy	days	17.3 ± 9.1	28.9 ± 32.6	0.03
Complication Tracheostoma site	Yes	16 (22.9%)	5 (41.7%)	
Difficult airway	Yes	18 (25.7%)	7 (58.3%)	0.02
Oral intake after surgery	days	14.8 ± 6.7	16.4 ± 7.3	
Neck dissection	Yes	58 (82.9%)	9 (75%)	
Brown Classification				
	I	7 (10%)		
	Ic	1 (1.4%)	1 (8.3%)	
	II	14 (20%)	3 (25%)	
	IIc	3 (4.3%)	1 (8.3%)	
	III	39 (55.7%)	5 (41.7%)	
	IV	5 (7.1%)		
	IVc	1 (1.4%)		
Flap type				
	Fibula	65 (92.9%)	9 (75%)	
	DCIA	4 (5.7%)	3 (25%)	
	Scapula	1 (1.4%)		
Bone Segments	n	2.1 ± 0.8	2.1 ± 0.8	
Flap Revision	Yes	16 (22.9%)	2 (16.7%)	
Flap success	Yes	56 (80%)	9 (75%)	
Partial Flap Loss	Yes	26 (37.1%)	4 (33.3%)	
Donor Site Complication	Yes	24 (34.3%)	2 (16.7%)	
Flap Site Complication	Yes	44 (62.9%)	9 (75%)	
Delirium	Yes	13 (18.6%)	6 (50%)	0.02
Anaesthesia induction time	min	68.2 ± 23.8	68.3 ± 24.5	
Length of surgery	min	598.1 ± 149.3	523.3 ± 148.4	
Preoperative Radiotherapy	Yes	20 (28.6%)	5 (41.7%)	
Cumulative dose	Gray	64.6 ± 6.6	65.4 ± 3.1	
Normal ward	days	21 ± 9.2	14.8 ± 9.4	0.03
Intensive Care Unit	days	4.4 ± 3.9	9.7 ± 6	<0.001
Length of stay	days	25 ± 9.5	24.2 ± 8.6	
Postoperative ventilation time	days	2.7 ± 1.4	5.7 ± 2.2	<0.001
Ventilation > 48 h	y/n	26 (37.1%)	11 (91.7%)	<0.001
BMI	kg/m^2^	23.8 ± 4.5	24.2 ± 6.2	
Smoking	Yes	51 (72.9%)	9 (75%)	
Alcohol	Yes	43 (61.4%)	7 (85.3%)	
Coronary heart disease	Yes	8 (11.4%)	2 (16.7%)	
Hypertension	Yes	33 (47.1%)	7 (58.3%)	
Charlson Comorbidity Index	Points	3.6 ± 1.9	3.2 ± 2.3	
COPD	Yes	10 (14.3%)		
HAP	Yes	10 (14.3%)	4 (33.3%)	

**Table 3:** Patient and treatment characteristics associated with overall tracheotomy:.

		No Tracheotomy N= 41	Tracheotomy N = 82	*p*-Value
Age	years	52.9 ± 14.6	59.1 ± 11.1	0.01
Previous surgery head neck	Yes	33 (80.5%)	40 (48.8%)	
Diagnosis				0.03
	Tumor	19 (46.3%)	60 (73.2%)	
	RONJ	15 (36.6%)	16 (19.5%)	
	MRONJ	1 (2.4%)	2 (2.4%)	
	Osteomyelitis	5 (12.2%)	4 (4.9%)	
	Trauma	1 (2.4%)		
Neck dissection	Yes	22 (53.7%)	67 (81.7%)	0.001
Brown Classification				0.001
	I	14 (34.1%)	7 (8.8%)	<0.001
	III	11 (26.8%)	44 (55%)	0.04
Bone Segments	n	1.73 ± 0.8	2.1 ± 0.8	0.01
Flap success	Yes	39 (95.1%)	65 (79.3%)	0.02
Flap Site Complication	Yes	17 (41.5%)	53 (64.6%)	0.01
Length of surgery	min	446.8 ± 122	587.2 ± 150.6	<0.001
Preoperative Radiotherapy	Yes	27 (65.9%)	25 (30.5%)	
Normal ward	days	11.2 ± 4.8	20.1 ± 9.4	<0.001
Intensive Care Unit	days	3.9 ± 4.8	4.8 ± 4.4	0.05
Ventilation > 48 h	y/n	15 (36.6%)	37 (45.1%)	
Charlson Comorbidity Index	Points	2.2 ±1.9	3.6 ± 1.9	<0.001

**Table 4:** Multivariate analysis to identify predictors for ventilation > 48 h, hospital acquired pneumonia, elective and unplanned tracheotomy.

	Variable	Coding	*p*-Value	OR	CI
Ventilation > 48 h					
	Operation time	Ø min	0.005	1.004	1.001–1.007
	Difficult airway	y/n	0.02	2.869	1.161–7.088
	Elective tracheotomy	y/n	0.006	0.054	0.007–0.440
HAP					
	Intensive Care Unit	Ø days	0.001	1.268	1.101–1.461
	Induction time	Ø min	0.009	1.039	1.009–1.069
	Elective tracheotomy	y/n	0.047	0.32	0.104–0.983
Elective Tracheotomy					
	Preoperative Radiation	y/n	0.018	2.81	1.184–6.257
	Charlson Comorbidity Index	Ø points	0.017	1.325	1.052–1.668
	Brown Class III	y/n	0.038	2.646	1.055–6.637
Unplanned Tracheotomy					
	Difficult airway	y/n	0.03	4.711	1.145–19.389
	Delirium	y/n	0.01	6.761	1.594–29.229

HAP: hospital acquired pneumonia.

## Data Availability

Data can be obtained by scientists that conducted the work independently from the industry, on request. Data are not stored on publicly available servers.
